# Risk factors of the stigma towards psychiatric patients among primary healthcare workers in China: a county study

**DOI:** 10.1186/s12888-017-1215-4

**Published:** 2017-02-08

**Authors:** Yang Wang, Xiaohua Wang, Weijun Zhang, Xiaoyun Liang, Donghua Tian, Zhiyong Qu

**Affiliations:** 1 School of Social Work, University of Illinois at Urbana-Champaign, 1010 W Nevada St, Champaign, 61801 IL USA; 2 School of Social Development and Public Policy, China Institute of Health and Center for Behavioral Health, Beijing Normal University, No. 19, XinJieKouWai St, Beijing, 100875 China

**Keywords:** Stigma, Primary healthcare, Mental health

## Abstract

**Background:**

Attitude towards psychiatric patients among healthcare workers has an impact on quality of medical care and rehabilitation of patients. In China, primary healthcare workers play an important role in mental health care, but little is known about the attitude of them towards psychiatric patients. This study aims to examine the risk factors associated with stigma among primary healthcare workers in West China.

**Methods:**

This cross-sectional study randomly recruited 395 primary healthcare workers in Mianzhu County, China. Data were collected via self-reported questionnaires. Descriptive analyses, bivariate analyses, and hierarchical linear regressions were performed by SPSS 17.0 to test the factors that accounted for the variation of stigma towards psychiatric patients.

**Results:**

Several risk factors were confirmed, including the satisfaction of income, work experience in psychiatric/ psychological departments, rehabilitation of patients, contact quality, and the attitude of mass media. However, demographic factors, the rest of work-related factors, and contact frequency might not be related with primary healthcare workers’ attitude towards psychiatric patients.

**Conclusions:**

The findings suggested that the quality of contact between primary healthcare workers and psychiatric patients be enhanced in order to decrease the stigma of healthcare staff. On-the-job training and institutional medical education needs a further exploration and development.

## Background

The number of psychiatric patients in China has been increasing fast and climbed to over 100 million. Over 16 million were diagnosed with severe psychiatric illness. Psychiatric illness has a negative impact on patients’ quality of life, and it lays a heavy burden on patients’ family [1–4].

The attitude of healthcare workers towards psychiatric patients and the improvement of patients’ situation can be casually linked. That is, healthcare workers’ attitude had a direct impact on patients’ behavior of seeking medical care, the quality of medical care, and patients’ rehabilitation. Further, it may influence the public’s attitude towards psychiatric patients as well [5, 6].

Among healthcare workers, a series of work-related variables have been demonstrated to associate with stigma, such as years of service [7], occupations [8], and income level [9]. Based on that, it is reasonable to assume that satisfaction level of income and working environment may also affect the attitude of primary healthcare workers’ attitude towards psychiatric patients. Moreover, treatment outcome could also contribute to stigma level as well. If the patients’ situation got improved after therapy, the stigma level of medical staff might get lower. On the contrary, if the treatment was ineffective, the stigma level could increase [8, 10]. Additionally, the understanding about the causes of mental illness was also proved related with stigma level. Those who believed that gene and physiological factors had a greater impact on mental illness than social environment and interaction did held a lower stigma level [11].

Early study demonstrated that stigma is formed through direct contact with the discriminated group, social interaction with other groups, and influence from mass media [12]. In the specific context of this study, the discriminated are patients with psychiatric illness. Some scholars believed that people would develop a better understanding of patients if they had an enhanced contact with psychiatric patients, and their stigma level would be lowered. Oppositely, people having no or little experience of communication with psychiatric patients would hold a negative attitude [9, 13–15]. In the aspect of social interaction, people whose relatives or friends had mental health issues would not fear psychiatric patients as much as people who had no such relatives or friends [16]. On the other hand, the attitude of family members and friends might also contribute to medical staff’s stigma. Specifically, in traditional Asian communities, collectivism occupies a dominant position [17]. Thus, the attitude of one’s significant others can also influence one’s own attitude toward a specific group. Mass media was another main source that one’s knowledge of psychiatric patients and mental issues came from. When more positive reports about patients were revealed to society, the stigma level of the public would tend to decrease. Inversely, if patients were described as a negative image to the public, the stigma level of the public might increase [4, 18, 19].

In China, primary healthcare workers consist of the healthcare workers in township clinics and communities. Although embedding service in communities has been advocated among BRICS countries [20], they are still playing the main role in delivering most of the mental health care service to community residents. Their service included giving regular wellness visits and directing outpatients to care service. However, most studies on medical staff’s attitude towards psychiatric patients were conducted among nurses in hospitals, healthcare workers in non-psychiatric departments, and healthcare workers in psychiatric department. For the studies conducted in township clinics, they just explored the knowledge level of mental illness among healthcare workers [21–24]. Therefore, little is known about the attitude of Chinese primary healthcare workers.

The purpose of this study was to examine the risk factors of stigma towards psychiatric patients among primary healthcare workers. We had the hypothesis that, socio-demographic and work-related factors, direct contact, social interaction, and mass media would influence the attitude of primary healthcare workers towards psychiatric patients. The findings of the study can provide a base to anti-stigma intervention among healthcare workers in future.

## Methods

### Participants and sampling

This study is a cross-sectional survey, using self-completion questionnaires. It was conducted in November 2013 in Mianzhu County, a county located in West China. Of the 24 towns in Mianzhu County, we randomly chose 12 towns. Then the primary healthcare workers in the 12 towns were investigated in this study. Eligible participants for this study were those who worked in township or village clinics. The study protocol was explained to them before the investigation, and those who had difficulty in reading or understanding the questions were excluded. The healthcare workers who accepted the investigation completed the questionnaires in their own clinics.

A total of 444 individuals participated the survey and returned the questionnaires. 395 of them (89.0%) completed the survey and were included into the final sample. Forty-nine individuals were excluded because they did not finish the scale which tested stigma level.

### Measures

- Demographics and work-related characteristics
  Participants offered the information about age, gender, religious belief, education level, and type of residence (hukou). In terms of work-related characteristics, they offered the information of their position, length of service, title, monthly income, satisfaction about income, and satisfaction about working environment. Two questions were about their evaluation of work. “What are the chances for patients to rehabilitate through the work of primary healthcare staff” (1 = large; 2 = general; 3 = small), and “How many patients you know have rehabilitated or stayed well” (1 = most of them; 2 = nearly half of them; 3 = fewer than half of them). Three other questions were about work and education experience: “Do you have work experience in psychiatric/psychological departments” (1 = yes; 2 = no), “Have you received training about mental health since work” (1 = yes; 2 = no), and “Have you received any formal education in medical school” (1 = yes; 2 = no).
- Stigma level
  Respondents’ stigma level towards psychiatric patients was assessed using Assessment Scale of Discrimination against Patients with Mental Disorders [25]. It was developed to assess the stigma level of Chinese healthcare workers in psychiatric and non-psychiatric departments, and relatives and friends of psychiatric patients with high level of reliability and validity [26, 27]. It contains 26 5-point questions, and it assesses the attitude in 3 dimensions: psychiatric patients’ social communication (e.g., “Psychiatric patients can become trustable friends”), psychiatric patients’ danger (e.g., “Many psychiatric patients committed crimes”), and psychiatric patients’ capability (e.g., “Psychiatric patients cannot adapt to school life”). For each item, the participant is required to choose the degree they agreed to. This study used the average score of questions in analyses. Higher scores indicate higher levels of stigma. In this study, its Cronbach’s alpha coefficient was 0.9, indicating a good reliability.
- Causes of psychiatric illness
  Eight 6-point questions were extracted from the Mental Health Locus of Origin (MHLO) scale [28]. This instrument has five items that attribute mental issues to “endogenous” factors (e.g., “The cause of most psychological problems is to be found in the brain”), which emphasizes the effect from gene and physiological factors. 3 items attribute it to “interactional” factors (e.g., “The mental illness of some people is caused by abuse or neglect during childhood”), which emphasizes the effect from social environment and interaction between people. The total score of the scale ranges from 0 to 80. A higher total score indicates that the respondent believes that the causes of psychiatric illness are more likely to be “endogenous,” We used the average score of the instrument in analyses. A lower total score of respondents indicates they believed that psychiatric illness was “interactional,” The Cronbach’s alpha coefficient of this instrument was 0.45 in this study, which is relatively low.
- Quality of contact
  The quality of contact between healthcare workers and patients was measured by 6 items. Each of the items uses one word to describe the relationship between participants and patients, and their polar opposites (equal and unequal status, involuntary or voluntary, superficial or intimate, pleasant or unpleasant, cooperative or competitive, and positive or negative). The words are placed as anchors on a 10-point scale. The overall quality is the average score of the scores of the 6 items. We used the average score of the instrument in analyses. A higher average score stands for a higher quality of contact [29]. The Cronbach’s alpha coefficient of this instrument was 0.64 in this study, which is good.
- Frequency of contact
  Contact Report was employed to measure the contact frequency between respondents and psychiatric patients [30]. This instrument describes 12 relationships that the participant and psychiatric patients may be in, indicating 12 levels of familiarity that the participant has to psychiatric patients. The scores of the 12 statements range from 1 to 12 (e.g., the item “Never observed person with mental illness” scores 1. And the item “I have a serious mental illness” scores 12). Participants choose every statement that fit their situation. The final score is decided by the largest score of the statement that is chosen. A higher final score indicates a more frequent contact with the psychiatric patients. In this study, the coefficient of reproducibility was 0.91, indicating a good reliability.
- Social interaction and media
  One question was asked to assess the attitude of their relatives and friends towards psychiatric patients: “What is the attitude of your relatives and friends towards psychiatric patients” (1 = sympathetic; 2 = neutral; 3 = aloof). Another question was asked to assess the attitude of media: “What is the attitude of the mass media towards psychiatric patients” (1 = sympathetic; 2 = neutral; 3 = aloof).

### Statistical analysis

SPSS 17.0 (SPSS Inc, Chicago IL) was used for all statistical analyses. All statistical tests were two-sided, and *p* < 0.05 was set as the significance level. Descriptive statistics were performed to examine whether the pattern of stigma level would vary across staff with different socio-demographic and work-related characteristics, and in different contexts of social interaction and media. Number (n), percentage (%), mean score, and standard deviation (SD) were given. Bivariate analyses were performed to test if the attribution way of psychiatric issues, and direct contact with patients (quality of contact, and frequency of contact) were significantly associated with stigma level. Hierarchical regression analysis was employed to explore the effects of demographic and work-related factors, direct contact, social interaction, and media on stigma level. Only the variables which were proved significantly related with stigma in the former steps of analyses were put into regression analysis. Standardized estimate (β), P, R^2^, and⊿R^2^ were provided.

## Results

### Characteristics of subjects

The characteristics of the participants are shown in Tables 1 and 2. Among the 395 primary healthcare workers, 128 were male (32.6%), 265 were female (67.4%). The age of 27 and 37 divided them into 3 groups with almost equal numbers. 84 of them had faith in Buddism (21.3%). Over half of them were urban residents (53.6%). Over half of them had an educational level higher than junior college (58.4%). Female healthcare workers showed a higher level of stigma than male participants (*p* < 0.01). Older healthcare workers showed a higher level of stigma relative to the younger ones (*p* < 0.01). Healthcare workers who were rural residents showed a higher level of stigma than the urban residents (*p* < 0.01). Compared with those variables, the participants’ educational level and faith in Buddhism were not significantly related with the participants’ stigma level.

**Table 1:** Numbers and percentages of the primary healthcare staff by basic characteristics and work-related characteristics

Variables	*n* (%)	Score (s.d.)	t/F	Variables	*n* (%)	Score (s.d.)	t/F
*Basic characteristics*							
Gender				Type of residence			
Men	128 (32.6)	1.82 (0.587)	−3.417**	Urban	210 (53.6)	1.90 (0.587)	2.491**
Women	265 (67.4)	2.03 (0.562)		Rural	182 (46.4)	2.04 (0.560)	
Age				Educational level			
18–27	123 (32.8)	2.07 (0.553)	3.375**	≤ High school	46 (11.9)	1.96 (0.469)	0.220
28–37	129 (34.4)	1.95 (0.611)		Secondary school	115 (29.7)	1.99 (0.581)	
≥ 38	123 (32.8)	1.89 (0.525)		≥ Junior college	226 (58.4)	1.95 (0.603)	
Buddhist							
Yes	84 (21.3)	2.06 (0.633)	−1.679				
No	311 (78.7)	1.94 (0.558)					
*Characteristics about occupations*							
Occupation				Monthly personal income (RMB, yuan)			
Doctor	101 (25.6)	1.97 (0.565)	5.826**	≤ 1000	134 (33.9)	1.99 (0.637)	6.041**
Nurse	94 (23.8)	2.19 (0.557)		1001–2000	104 (26.3)	2.05 (0.527)	
Other staff in township clinics	59 (14.9)	1.89 (0.617)		> 2000	64 (16.2)	1.74 (0.534)	
Village doctor	95 (24.1)	1.88 (0.563)					
Length of service				Satisfaction about income			
1–5 years	116 (29.4)	2.05 (0.555)	2.531	Satisfied	72 (18.2)	1.79 (0.516)	0.422**
6–15 years	106 (26.8)	2.06 (0.590)		General	185 (46.8)	1.94 (0.557)	
≥ 16 years	112 (28.4)	1.90 (0.597)		Unsatisfied	124 (31.4)	2.09 (0.629)	
Title				Satisfaction about work environment			
None	141 (35.7)	2.02 (0.572)	2.165	Satisfied	124 (31.4)	1.79 (0.565)	9.803***
Physicians’ assistant	52 (13.2)	1.83 (0.537)		General	241 (61.0)	2.02 (0.573)	
Primary/intermediate/advanced title	179 (45.3)	1.97 (0.592)		Unsatisfied	26 (6.6)	2.20 (0.445)	

**P < 0.01, ***P < 0.001

**Table 2:** Numbers and percentages of the primary healthcare staff by characteristics about evaluation of work, knowledge of psychiatric illness, and social culture

Variables	*n* (%)	Score (s.d.)	t/F	Variables	*n* (%)	Score (s.d.)	t/F
*Evaluation of work*							
The chances for patients to rehabilitate through the work of primary healthcare staff				Patients known to have rehabilitated or stayed well			
Large	173 (43.8)	1.85 (0.552)	6.893**	Most of them	164 (41.5)	1.83 (0.608)	14.894***
General	170 (43.0)	2.03 (0.597)		Nearly half of them	114 (28.9)	1.94 (0.551)	
Small	44 (11.1)	2.15 (0.461)		Fewer than half of them	108 (27.3)	2.21 (0.489)	
*Knowledge of psychiatric illness*							
Work experience in psychiatric/psychological departments				Training about mental health since work			
Yes	33 (8.4)	1.71 (0.599)	2.637**	Yes	230 (58.2)	1.96 (0.566)	0.312
No	354 (89.6)	1.99 (0.575)		No	157 (39.7)	1.98 (0.592)	
Medical school education							
Yes	328 (83.0)	1.95 (0.600)	1.045				
No	50 (12.7)	2.04 (0.482)					
*Social culture*							
Attitude of relatives and friends towards patients				Attitude of mass media towards patients			
Sympathetic	233 (59.0)	1.86 (0.601)	11.227***	Sympathetic	283 (71.6)	1.91 (0.588)	7.730**
Neutral	111 (28.1)	2.13 (0.462)		Neutral	72 (18.2)	2.02 (0.467)	
Aloof	43 (10.9)	2.13 (0.533)		Aloof	32 (8.1)	2.31 (0.516)	

**P < 0.01, ***P < 0.001

### Correlations among other variables

Result of the relation analyses of work-related variables is presented in Table 2. Compared with other staff, nurses tended to hold more stigmatizing attitudes. Less monthly income, less satisfied to income, and less satisfied to working environment indicated a higher level of stigma, too. Those who believed the chances patients rehabilitated through their work was smaller, those who knows fewer patients that rehabilitated or got better, and those who did not have work experience in psychiatric/psychological departments held a higher level of stigma. Those whose relatives and friends were aloof to patients, and those who believed that mass media held a aloof attitude towards patients also had a higher level of sigma (all *p* values <0.01). On the contrary, length of service, title, on-the-job training experience, and medical school education experience were not significantly related with the participants’ stigma level.

Table 3 reveals that the participants’ contact quality with patients was significantly related with stigma level (*p* < 0.01). Those who had higher contact quality tended to have a lower stigma level. By comparison, the quantity of contact and the attribution of psychiatric illness did not show a significant relationship with the stigma scores.

**Table 3:** Bivariate correlate analysis of stigma level, quality of contact, quantity of contact, and causes of psychiatric illness

	Stigma level	Quality of contact	Quantity of contact	Causes of psychiatric illness
Stigma level	1			
Quality of contact	−0.374**	1		
Quantity of contact	−0.01	−0.031	1	
Causes of psychiatric illness	0.013	−0.138**	−0.06	1

**P < 0.01

### Hierarchical linear regression analyses

The results of the hierarchical regression analyses were shown in Table 4. Because of the significant effects of gender, age, and type of residence, those variables were included into Model 1 where stigma level was the dependent variable. And Model 1 just accounted for 3.5% of the variance in stigma. As demonstrated in Model 2, with work-related variables, contact quality, and the demographic variables, Model 2 as a whole accounted for 29.5% of the variance in stigma.

**Table 4:** Hierarchical linear regression analysis of the influence of demographic factors, work-related factors, contact quality, social interaction, and media on the stigma of primary healthcare workers

		Model 1		Model 2		Model 3	
		β	*P*	β	*P*	β	*P*
Gender	Male: Female	−0.161	0.018	−0.055	0.432	−0.055	0.425
Type of residence	Urban: Rural	−0.044	0.507	−0.053	0.426	−0.051	0.445
Age	Ref: 18–27						
	28–37	−0.013	0.869	−0.060	0.399	−0.082	0.245
	≥37	0.04	0.616	−0.020	0.812	−0.050	0.543
Work experience in psychiatric/psychological department	Yes: No			−0.172	0.006	−0.174	0.006
Occupation	Ref: Village doctors						
	Doctors			0.012	0.883	−0.001	0.989
	Nurses			0.134	0.130	0.078	0.385
	Other staff in township clinics			−0.077	0.290	−0.073	0.313
Monthly personal income (RMB, yuan)	Ref: ≥2000						
	0–1000			−0.040	0.677	−0.012	0.899
	1001–2000			−0.026	0.774	−0.002	0.979
Satisfaction about income	Ref: Low						
	High			−0.037	0.606	−0.05	0.490
	Medium			−0.207	0.003	−0.187	0.007
Satisfaction about working environment	Ref: Low						
	High			−0.174	0.147	−0.111	0.352
	Medium			−0.095	0.404	−0.046	0.686
The chances for patients to rehabilitate through the work of primary healthcare staff	Ref: Low						
	High			−0.036	0.734	0.046	0.667
	Medium			0.030	0.768	0.074	0.460
Patients known to have rehabilitated or stayed well	Ref: fewer than half						
	Most			−0.117	0.158	−0.139	0.092
	Nearly half			−0.160	0.030	−0.183	0.012
Quality of contact				−0.343	0.000	−0.313	0.000
Attitude of relatives and friends towards patients	Ref: Aloof						
	Sympathetic					−0.182	0.061
	Neutral					−0.050	0.603
Attitude of mass media towards patients	Ref: Aloof						
	Sympathetic					−0.218	0.020
	Neutral					−0.243	0.007
R^2^		0.035		0.295		0.333	
⊿R^2^		0.035		0.260		0.038	

After including all of the factors, the final results of Model 3 showed that model could account for 33.3% of the variance in stigma. Those who had no work experience in psychiatric/psychological departments (β = −0.174, *p* = 0.006), those who knew fewer than half of patients rehabilitated (β = −0.183, *p* = 0.012), those who had a medium level of satisfaction of income (β = −0.313, *p* = 0.000), those who had a lower contact quality (β = −0.218, *p* = 0.020), or those who were exposed to mass media with an aloof attitude (β = −0.243, *p* = 0.007) were revealed to have an significantly higher level of stigma (F = 4.747, *p* < 0.001). Table 4 also showed that the values of ⊿R^2^ of Model 2 to Model 3 were 0.260 and 0.038 respectively.

## Discussion

As to our knowledge, this study is one of the first studies focusing on the risk factors of stigma towards psychiatric patients among primary healthcare workers in China. This study found that stigma level was associated with contact quality and the attitude held by mass media. Moreover, satisfaction level of income, work experience in psychiatric/ psychological departments, and the rehabilitation of patients were also significantly related with stigma level.

### Work-related factors

Previous studies had revealed that only a small proportion of Chinese village doctors had received formal medical school education [31–36]. However, a significant difference in stigma between doctors in village clinics and healthcare workers township clinics did not exist in this study. That result can be interpreted partially in light of work experience [7]. The proportion of village doctors who had over 16 years’ work experience was 59.1%, which is much higher than that of township clinic doctors (35.2%), nurses (13.5%), and other staff (20.0%). Moreover, most of the village doctors work in the same villages in which they were born and grew up, so they have possibly built a close and stable relationship with villagers, including psychiatric patients. Thus, it was their work and life experience that made them not have a higher level of stigma than the rest of the participants.

In terms of the knowledge level of the participants, for the healthcare workers who had work experience in psychiatric/psychological departments of the primary healthcare institutions, most of the patients they served were at recovery phase, and that contributed to the formation of their tolerant attitude and lower level of stigma. That is in accordance with Patten’s study [37]. On the contrary, the group who had medical school education and the group who had not did not have a significant difference in stigma level. That finding reflected the lack of humanistic education in medical school education. In China, most of the medical schools did not have enough medical humanity education, and that led to the unbalanced development of medical students’ medical skill and the capability to be empathetic, and this influence will continue to exist when they go to work. And it might cause the tense relationship between healthcare workers and patients [34]. On-the-job training has been a necessity in achieving integrated primary care for mental health [38]. But this current study did not show that receiving on-the-job training was associated significantly with a lower stigma level. The insignificant association could be explained by the factors that had an impact on the effectiveness of training. They can be attendance, the content of training, and the capability of trainers [39]. Therefore, more detailed information on training is necessary to make an improvement in training.

Mbanya et al. found that most nurses agreed that a better salary was a good incentive to give a better service with humanity [40]. In contrast, results of this study showed that monthly income did not prove to be related with stigma level significantly. Whereas the results showed that those who were unsatisfied about income had a significantly higher stigma level than those who had a medium level of satisfaction. That finding reflected that apart from income itself, other factors such as family size, consumption, work load, and colleagues’ income could also influence participants’ financial situation and satisfaction about their income, and that would influence their mood at work as a result [41].

### Direct contact

Additionally, healthcare workers who had a higher quality of contact, which indicated that those who were more voluntary, pleasant, positive, and treated patients as equal, had a significantly lower level of stigma. On the contrary, quantity of contact with patients did not prove a tendency of variance. This is in accordance with previous studies, which emphasized that it was the nature of contact that explained the variance of stigma towards mental illness [42, 43].

### Social interaction and media

The attitude held by relatives and friends did not have a significant relationship with that of the participants. It was unexpected because it was acknowledged that Asian traditional communities were characterized by collectivism, and community members would always follow the social norms [17, 44]. In addition, most of the village doctors in our sample were born and worked in the same villages. Thus, it is reasonable to state that the mechanism through which social interaction worked to shape the individual’s attitude is more complex and it needs further explorations. Other factors about the community, such as community’s locality and population size, should have been included, because those factors can shape characteristics of the residents’ interaction [45, 46]. The attitude of mass media showed a significant relationship with stigma level, and that is consistent with findings of previous studies [4, 19].

### Limitations

Several limitations should be acknowledged when interpreting the findings. First, the cross-sectional design made the study cannot generate a causal relationship between the diverse factors and stigma level. Second, this study was conducted in just one county in West China. As China’s health care resources have a mal-distribution, the results of this study does not have a strong generality to other areas of China [47]. A third limitation is the instruments we employed in assessment. In the case of Contact Report, some of its items were just inquired by asking whether the events had happened to the interviewees, without digging deeper for the accurate frequency of the events. For example, for the item “Provides service to persons with mental illness”, we could not tell how many patients they had served, and what kind of service they had provided from the answer. Thus, respondents with the same final score of this scale still could be much different in their contact frequency with patients. That might lead to the insignificant relationship between quantity and stigma level, and it could be the same with other variables. Future studies are expected to have a more accurate measurement.

Moreover, we only included two questions to assess the characteristics of social interaction and media. That may have led to a biased conclusion. Future studies should do more to supplement the measurement in the two dimensions.

### Implications

Psychiatric care in primary healthcare is the first line of care, and it can be effective. However, its importance is always overlooked [48]. Reducing stigma is necessary when integrating mental health care into primary health care [38]. The current study advances the literature by highlighting the factors which can impact the stigma level towards psychiatric patients among primary healthcare workers. In order to extend the research, future studies should make a further exploration to the characteristics of specific groups of primary healthcare workers. For example, it’s found that village doctors were always older than other groups [49], which means the medical education they received can be quite different from that received by the younger health workers.

The outcome of the current study has implications for the points on which intervention programs aiming at reducing the stigma held by primary healthcare workers should focus. First, enhance the communication between healthcare workers and psychiatric patients, and between experienced and young healthcare workers. Second, foster medical humanity in course-based institutional education and provide well-designed on-the-job training programs [50]. Third, make better use of the present health human resources. The significance of incorporate mental health care had been emphasized by previous studies, and healthcare staff in different positions are supposed to build up effective communication and teamwork to deliver more effective primary mental health care [51, 52]. Even in the brief communication between medical staff from different positions, the work experience of senior healthcare workers can help the younger ones to develop a more positive attitude towards patients. Finally, mass media should propagate mental health knowledge to help create a more friendly and receptive society to psychiatric patients.

## Conclusions

For Chinese primary healthcare workers, their stigma level towards psychiatric patients is associated with their work-related characteristics, direct contact with patients, social interaction, and media. Those who were with a medium level of satisfaction towards income, who had work experience in psychiatry/psychological departments, who knew more recoveries of psychiatric patients, and who had a higher contact quality held a significantly lower level of stigma than the others. And the attitude of mass media was significantly related with stigma level as well. However, the limitation in generality of the result needs to be addressed in future studies.
